# Protective effect of thymoquinone against lung intoxication induced by malathion inhalation

**DOI:** 10.1038/s41598-021-82083-w

**Published:** 2021-01-28

**Authors:** Walied Abdo, Mostafa A. Elmadawy, Ehab Yahya Abdelhiee, Mona A. Abdel-Kareem, Amira Farag, Mohamed Aboubakr, Emad Ghazy, Sabreen E. Fadl

**Affiliations:** 1 Pathology Department, Faculty of Veterinary Medicine, Kafrelsheikh University, Kafr Elsheikh, 33516 Egypt; 2 Forensic Medicine and Toxicology Department, Faculty of Veterinary Medicine, Kafrelsheikh University, Kafr Elsheikh, 33516 Egypt; 3 Forensic Medicine and Toxicology Department, Faculty of Veterinary Medicine, Matrouh University, Matrouh, Egypt; 4 Anatomy and Embryology Department, Faculty of Medicine, Kafrelsheikh University, Kafr Elsheikh, 33516 Egypt; 5 Department of Pharmacology, Faculty of Veterinary Medicine, Banha University, 13736 Moshtohor, Toukh, Qaliobiya Egypt; 6 Clinical Pathology Department, Faculty of Veterinary Medicine, Kafrelsheikh University, Kafr Elsheikh, 33516 Egypt; 7 Biochemistry Department, Faculty of Veterinary Medicine, Matrouh University, Matrouh, Egypt

**Keywords:** Biochemistry, Biological techniques, Systems biology

## Abstract

Malathion is considered one of the vastest pesticides use all over the world. Malathion-inhalation toxicity commonly occurred in many occupational farmers. Therefore, this study aimed to ameliorate the possible malathion-induced pulmonary toxicity through thymoquinone administration. Forty animals were used to conduct our study, divided into five groups; G1 control group, G2 thymoquinone (50 mg/kg) group, G3 malathion group (animals inhaled 100 mg/ml/m^3^ for 15 min for 5 days/week for three weeks), G4 and G5 were subjected to the same malathion inhalation protocol beside oral thymoquinone administration at doses of 25 and 50 (mg/kg), respectively. Malathion-inhalation induced marked systemic toxicity as hepatotoxicity and nephrotoxicity associated with increased serum hepatic and renal enzymes, and hypersensitivity accompanied with increased total IgE serum level. The lung showed severe interstitial pneumonia associated with severe vascular damage and marked eosinophil infiltration. Moreover, the lung showed a marked decrease in the pulmonary surfactant protein, especially SP-D gene expression. While, thymoquinone treatment to malathion-inhaled animals decremented the following; hepatic enzymes and renal function tests, total IgE as well as pneumonia and hypersensitivity pathological features, and augmented the expression of SP-D. In conclusion, thymoquinone could be potentially used in pest control workers to ameliorate the systemic and pulmonary intoxication caused by one of the most field-used pesticides.

## Introduction

In agriculture, pesticides have been used in the eradication of insects and controlling vectors of the diseases^1^. About three million poisoning cases and more than 250,000 deaths occur due to pesticides every year^2^, where the most common pesticides are organophosphates. Organophosphates pesticides (OPs) lead to environmental pollution, causing great public concern^1^. Moreover, some OPs can be detected as residue in the soil, food, and food products^3^. So, the human body exposes to pesticides in three ways; dermal and oral in all humans or inhalation by agriculture workers, especially in countries that do not use pesticide safety measures^4^. When toxic amounts of OPs are inhaled, their first symptoms are usually a respiratory system with a bloody or runny nose, and wheezing in bronchial tubes because of constriction or excess of fluid^5^. The frequently and widely used organophosphate is malathion (*S*-1,2(bis-ethoxycarbonyl)ethyl-*O,O*-di-methyl phosphorodithioate) with high toxicity^1–6^. Depending on the above-mentioned information, the metabolic and pathological adverse effects of malathion inhalation were studied in rats. Where Ruckmani et al.^7^ reported that malathion inhalation adversely affects blood glucose. On the other hand, Hectors et al.^8^, Rezg et al.^9^, and Beard et al.^10^ recorded its adverse effects on the metabolism of carbohydrate and lipid, immune, and nervous systems. Malathion adversely affects the liver, kidney, and hematological parameters^11^. Histopathologically, malathion increases alveolar macrophages and interstitial neutrophil infiltration and minimal fibrosis^1^.

Thymoquinone (TQ) is the primary ingredient (approximately 30–40%) of black seed oil (Nigella sativa) ^12^, and is a bioactive monomer, chemically is 2-methyl-5-isopropyl-1, 4-benzoquinone^13,14^. N. sativa is a plant commonly used for the treatment of various diseases, such as high blood sugar levels, asthma, and eczema in Arabian countries^15^. TQ characteristic in modern pharmacology varies as it contains active Quinone and has a range of therapeutics effects through anti-inflammatory and analgesic activities ^16^. Also, Abdel-Daim et al.^17^ reported that TQ enhances the biochemical and oxidative impacts of malathion, likely by reducing reactive oxygen and nitrogen radicals. Despite current research with animals, it is difficult to predict the degree that pesticide exposure by inhalation affects humans because the rats are resistant rather than human. This protocol examined the possible protective role of different doses of TQ against pulmonary, renal, and hepatic toxicity of malathion inhalation in rats through estimation of some serum biomarkers, pathology, gene expression, and immunohistochemistry.

## Material and methods

### Chemicals

Malathion (high technical grade of 98% active ingredient was obtained from El- Nasr Co. for Intermediate Chemicals (Abou Rawash, Giza, Egypt), and thymoquinone from Sigma Al-drich Co., USA (CAS Number 490–91-5; purity ≥ 98%). All biochemical analysis kits were purchased from Biodiagnostics Company (Dokki, Giza, Egypt).

### Animals and experimental protocols

**Figure 1:**
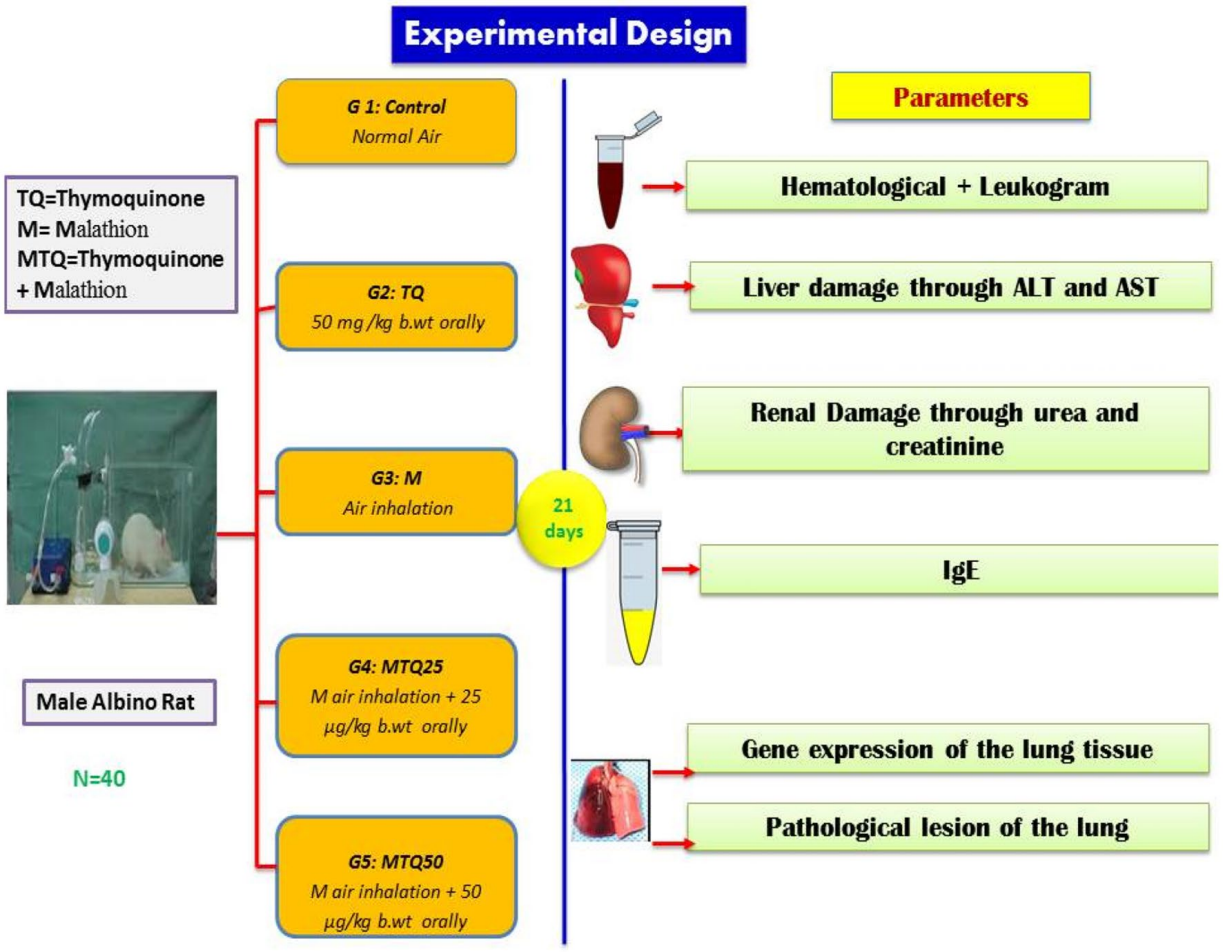
Protocol of the experiment.

Forty Sprague–Dawley male rats (220–250 g) were purchased from the laboratory animal house, Faculty of Agriculture, Kafrelsheikh University. Laboratory animals were housed in plastic cages in an air-conditioned room with a 12 h dark/light cycle and temperature 25 ± 2 °C and received standard laboratory balanced commercial diet and water ad libitum. The rats were kept for one week (adaptation period) before starting the experiment. The adapted laboratory animals were divided into (Fig. 1) five groups (8 rats per each group) as following; control group (G1) was given 1 ml of normal saline orally by gastric tube and received vehicle distilled water aerosol, thymoquinone group (G2) was given 1 ml of thymoquinone solution at a dose (50 mg/kg) orally by gastric tube^18^ and received vehicle distilled water aerosol, malathion group (G3) inhaled 1000 mg /m^3^ (1/5 of inhalation LC_50_), (reference is Material Safety Data Sheet, Malathion (2011) 500E, PCP # 4709. Dorchester, Ontario, Canada: Registration and Regulatory) for 15 min for 5 days/week for three weeks) and administered 1 ml of normal saline through the oral route, and the other two groups (G4 and G5) were also inhaled malathion and then given thymoquinone orally at two levels of dosing 25 mg and 50 mg/kg body weight for G4 and G5, respectively. Control and sham groups had a separate cage to avoid possible residues of malathion due to routine successive use. All animal handling procedures are in agreement with the ARRIVE guidelines from the National Center for the Replacement, Refinement, and Reduction of Animals in Research (NC3Rs)^19^ along the experimental period (21 days). The experimental protocol was accepted at Kafrelsheikh University, Egypt, by the institutional animal care and use committee 33516.

### Sample preparation for hematological and biochemical parameters

At the end of the protocol (after 21 days), the rats were anesthetized by isoflurane then punctured retro-orbital plexus for collection of blood samples. The blood samples were collected on an anticoagulant for hematological and leukogram estimation. For serum biochemistry, the blood samples were collected without anticoagulant for serum separation. Haemtological parameters were performed by automatic Exigo vet 400 coulter (Boule Diagnostics AB, Domnarvsgatan, Spånga, Sweden). The activities of the serum enzymes (alanine aminotransferase (ALT), aspartate aminotransferase (AST), and alkaline phosphatase (ALP)) and serum concentration of urea and creatinine were estimated spectrophotometrically according to the instructions of Bio-Diagnostic Company research kits (Giza, Egypt). Meanwhile, the total IgE level was analyzed by a sandwich ELISA kits at 450 nm wavelength (CUSABIO Technology LLC, Houston, TX 77054, United States).

The rats from different experimental groups were anesthetized by isoflurane and used for blood sample collection then euthanized using an intraperitoneal injection of an overdose of pentobarbital anesthesia (300 mg/kg). The remaining live rats were euthanized in strong bags by CO_2_ suffocation. All dead rats and remnants of samples and bedding material, were buried in the strict hygienically controlled properly constructed burial pit.

### Histopathology

The lung samples of different animal groups were trimmed, fixed in neutral buffered formalin (10%), dehydrated, cleared, and embedded in paraffin wax. The hard paraffin blocks were sectioned to make a ribbon of tissue (5–6 µm thicknesses). The tissue ribbon was mounted on a water bath, fixed on a glass slide, and further stained with hematoxylin and eosin (H&E)^20^. Histological examination was done using a Leica microscope. The quantitative assessment of the histopathological lesions was estimated within the animals of different groups. The lesions score was estimated according to nine points divided equally on the vascular lesions, pneumonia, and alveolar patency according to Yamanel et al.^21^ with some modifications. The vascular lesions varied from score 0, which revealed normal blood vessels, score 1 indicated congestion, score 2 indicated congestion, oedema, and hemorrhage, and score 3 indicated all the previous findings with marked loss of the tunica media of the blood vessels and extensive hemorrhage. Pneumonia score was assessed according to the following criteria; score 0 indicated normal lung, score 1 indicated mild interstitial pneumonia with the absence of the perivascular reaction, score 2 indicated a moderate degree of interstitial pneumonia with limited perivascular inflammatory cells infiltration, and score 3 indicated marked interstitial pneumonia associated with the marked perivascular reaction. The alveolar patency was classified into score 0 that indicated normal patent alveoli with thin interalveolar septa lined with type I and II alveolar cells, score 1 indicated mild thickening of the alveolar septa with patent alveoli, score 2 noted moderate thickening of the septa associated with decreasing the alveolar spaces and score 3 that revealed marked thickening of the connective tissue septa with marked decrease and sometimes showed focal obliteration of the alveoli.

### Immunohistochemistry of C-KIT and survivin

Survivin immunohistochemistry was performed according to Khalil et al.^22^. In brief, the lung tissues were mounted on positively charged slides, rehydrated, and then put in EDTA solution PH 8 for antigen retrieval. The slides were treated with 0.3% of hydrogen peroxide for 15 min and protein block solution for 30 min. The slides were then incubated with rabbit polyclonal C-KIT (Invitrogen, Carlsbad, CA, Cat. No 34-8800, 1:50 dilution) and polyclonal survivin antibody (Novus Biological LLC, Briarwood Avenue, Centennial, USA; Cat. No NB500-201; 1:400 dilution). The slides were rinsed three times with PBS and then incubated with anti-rabbit IgG secondary antibodies for 30 min (EnVision + System HRP; Dako). The slides were visualized with diaminobenzidine commercial kits (Liquid DAB + Substrate Chromogen System; Dako) and counterstained with Mayer's hematoxylin. The primary antibody was replaced by the normal mouse serum as a negative control slide. The labeling index of survivin was expressed as the percent of positive area/mm2 using ImageJ analysis, NIH, USA.

### Pulmonary surfactant protein D gene expression

The pulmonary tissue samples were collected from 5 laboratory animals from each group in clean Eppendorf tubes and stored at − 80 °C until used. RNA was extracted from the pulmonary tissues using the Qiagen RNeasy Plus Mini kit. Then, RNA concentration in each sample was detected by Nano Drop ND-1000 Spectrophotometer at 260 and 260/280 nm ratios (Nano Drop Technologies, Wilmington, Delaware, USA). The cDNA was developed from the RNA samples using Maxima First Strand cDNA Synthesis Kit (Thermo Fisher Scientific, USA). Amplification was done using the Thermo Scientific MAXIA SYBR Green/ROXqPCR Master Mix (2×). Gene-specific PCR primers^23,24^ were designed as mentioned in Table 1.

**Table 1:** Primers sequence used in our study.

Gene		Sequence (5′-3′)	References
SP-D	Forward	ACTCATCACAGCCCACAACA	(Tian et al.^23^)
	Reverse	TCAGAACTCACAGATAACAAG	
β-actin	Forward	AAGTCCCTCACCCTCCCAAAAG	(El-Magd et al.^24^)
	Reverse	AAGCAATGCTGTCACCTTCCC	

*SP-D* surfactant protein D, *β-actin* beta-actin.

### Statistical analysis

All values were expressed as mean ± SD. The data were analyzed by one-way ANOVA followed by Duncan’s post hoc test for multiple group comparisons using Graph Pad PRISM, software, La Jolla, CA, USA. https://www.graphpad.com. Variance was considered statistically significant when p ≤ 0.05.

## Results

### Hematological parameters

The results of the hemogram were statistically (P ≤ 0.05) decreased in RBCs count, Hb, HCT, MCV, MCH, MCHC, and PLT in the G3 group as a result of malathion inhalation compared to other groups (Table 2). Moreover, the above-mentioned parameters were improved with two levels of TQ supplementation in the malathion inhaled rats (G4 and G5) nearly to normal (G1). On the other hand, TQ alone not affect hemogram when compared with the control group (G1).

**Table 2:** Effect of thymoquinone supplementation and/or malathion inhalation on hemogram of rats.

Groups Parameters	G1	G2	G3	G4	G5
RBCs count (× 10^6^/µl)	7.25 ± 0.74^ab^	7.54 ± 0.29^a^	5.65 ± 0.39^c^	6.54 ± 0.26^b^	7.04 ± 0.21^ab^
Hb (g%)	14.56 ± 0.43^ab^	15.04 ± 0.52^a^	10.58 ± 0.55^c^	13.26 ± 0.49^b^	14.16 ± 0.41^ab^
HCT (%)	45.38 ± 0.86^ab^	46.34 ± 1.47^a^	40.74 ± 0.96^c^	43.04 ± 0.43^b^	44.38 ± 0.58^ab^
MCV (fl)	63.31 ± 0.78^a^	62.89 ± 1.29^a^	72.26 ± 1.69^b^	62.41 ± 1.14^a^	63.14 ± 1.07^a^
MCH (Pg)	19.51 ± 0.51^ab^	20.18 ± 0.22^a^	16.01 ± 0.42^c^	18.56 ± 0.34^b^	20.01 ± 0.50^a^
MCHC (g/dl)	31.77 ± 0.36^a^	32.22 ± 0.46^a^	25.81 ± 0.59^c^	30.14 ± 0.40^b^	31.33 ± 0.18^ab^
PLT (× 10^3^/µl)	363.88 ± 15.39^a^	370.25 ± 10.36^a^	267.75 ± 13.57^b^	329.88 ± 10.99^a^	351.50 ± 19.40^a^

Values are expressed as mean ± standard errors. Means in the same row (a-b) with different subscript letters significantly differ at (p ≤ 0.05).

*RBCs* red blood cells, *Hb* hemoglobin, *HCT* hematocrit, *MCV* mean corpuscular volume, *MCH* mean corpuscular hemoglobin, *MCHC* mean corpuscular hemoglobin concentration.

G1 = control group, G2 = thymoquinone group (50 mg/kg body weight), G3 = malathion group G4 = malathion inhalation + thymoquinone 25 mg/kg body weight, and G5 = malathion inhalation + thymoquinone 50 mg/kg body weight.

### Leukogram of the blood

Regarding the result of WBCs, there was a statistically increase in WBCs count in the G3 group compared to other groups (P ≤ 0.05). The leukogram demonstrated a noticeable decrease of LYM and an increase of MON, EOS, BAS, and NEU in the G3 group as a result of malathion inhalation compared to the other groups (P ≤ 0.05) (Table 3). Meanwhile, the supplementation of TQ at a dose of 50 mg/kg in the malathion inhaled rats improved rats' leukogram where the results of the leukogram return to normal except the result of WBCs was significantly (P ≤ 0.05) increased in G5 when compared with the control one. Moreover, TQ at a low dose (25 mg/kg) in the malathion inhaled rats significantly (P ≤ 0.05) increased all parameters of the leukogram except lymphocyte, which was significantly decreased when compared with the control group (G1). On the other hand, TQ alone not affected leukogram when compared with the control group (G1).

**Table 3:** Effect of thymoquinone supplementation and/or malathion inhalation on leukogram of rats.

Groups Parameters	G1	G2	G3	G4	G5
WBCs count (× 10^3^/µl)	9.68 ± 1.36^c^	8.67 ± 0.71^c^	26.84 ± 2.18^a^	18.74 ± 1.05^b^	15.17 ± 1.88^b^
LYM %	66.16 ± 5.33^ab^	69.75 ± 5.53^a^	32.78 ± 3.00^d^	50.84 ± 10.09^c^	62.92 ± 5.30^b^
MON %	3.5 ± 0.49^c^	3.4 ± 1.29^c^	8.19 ± 2.52^a^	5.11 ± 0.62^b^	4.48 ± 0.67^bc^
ESO %	2.98 ± 0.92^b^	2.49 ± 0.44^b^	10.43 ± 8.11^a^	5.29 ± 0.79^b^	4.08 ± 1.13^b^
BAS %	0.73 ± 0.39 cd	0.56 ± 0.42^d^	4.51 ± 0.94^a^	2.93 ± 0.27^b^	1.25 ± 0.46^c^
NEU %	26.64 ± 5.62^c^	23.80 ± 5.26^c^	44.10 ± 7.38^a^	35.84 ± 10.23^b^	27.28 ± 5.92^c^

Values are expressed as mean ± standard errors. Means in the same row (a-b) with different subscript letters significantly differ at (p ≤ 0.05).

*WBCs* white blood cells, *LYM* lymphocyte, *MON* monocyte, *ESO* eosinophil, *BAS* basophil, *NEU* neutrophil.

G1 = control group, G2 = thymoquinone group (50 mg/kg body weight), G3 = malathion group G4 = malathion inhalation + thymoquinone 25 mg/kg body weight, and G5 = malathion inhalation + thymoquinone 50 mg/kg body weight.

### Biochemical determination of the serum

The result of serum total IgE level was statistically increased in the G3 group compared with other groups (P ≤ 0.05) (Table 4). Moreover, the supplementation of a low level of TQ (25 mg/kg) in the malathion inhaled rats significantly increased serum IgE when compared with the control group. Meanwhile, the high level of TQ in malathion inhaled rats insignificantly increased serum IgE when compared with the control group. The effect of the serum biomarkers of the liver and kidney tissue damages showed a significant (P ≤ 0.05) increased in the activities of AST, ALT, and ALP, and concentration of urea and creatinine in the malathion group (G3). The adverse effects of malathion inhalation on immunity and serum biomarkers were improved by TQ supplementation in a dose-dependent manner (Table 4). On the other hand, TQ alone not affected serum biomarkers of the liver and kidney damage when compared with the control group (G1).

**Table 4:** Effect of thymoquinone supplementation and/or malathion inhalation on serum biochemistry of rats.

Groups Parameters	G1	G2	G3	G4	G5
IgE (ng/ml)	30.75 ± 7.59 cd	28.13 ± 6.83^d^	98.63 ± 9.61^a^	56.38 ± 7.09^b^	45.38 ± 7.42^c^
AST (U/L)	56.48 ± 1.49^c^	56.54 ± 2.25^c^	113.65 ± 3.08^a^	65.98 ± 4.55^b^	48.40 ± 2.26^c^
ALT (U/L)	39.12 ± 1.97^c^	31.28 ± 1.89^c^	85.22 ± 4.33^a^	55.58 ± 3.39^b^	39.35 ± 1.76^c^
ALP (U/L)	147.80 ± 6.39 cd	143.20 ± 7.21^d^	232.00 ± 6.32^a^	186.60 ± 4.68^b^	164.80 ± 5.79^c^
Urea (mg/dl)	25.99 ± 2.04^d^	29.93 ± 1.68 cd	59.08 ± 3.95^a^	44.60 ± 2.81^b^	34.89 ± 1.65^c^
Creatinine (mg/dl)	0.93 ± 0.04^c^	0.94 ± 0.06^c^	1.66 ± 0.09^a^	1.31 ± 0.03^b^	1.17 ± 0.05^b^

Values are expressed as mean ± standard errors. Means in the same row (a-b) with different subscript letters significantly differ at (p ≤ 0.05).

*AST* aspartate aminotransferase, *ALT* alanine aminotransferase, *ALP* alkaline phosphatase.

G1 = control group, G2 = thymoquinone group (50 mg/kg body weight), G3 = malathion group G4 = malathion inhalation + thymoquinone 25 mg/kg body weight, and G5 = malathion inhalation + thymoquinone 50 mg/kg body weight.

### Histopathological findings

**Figure 2:**
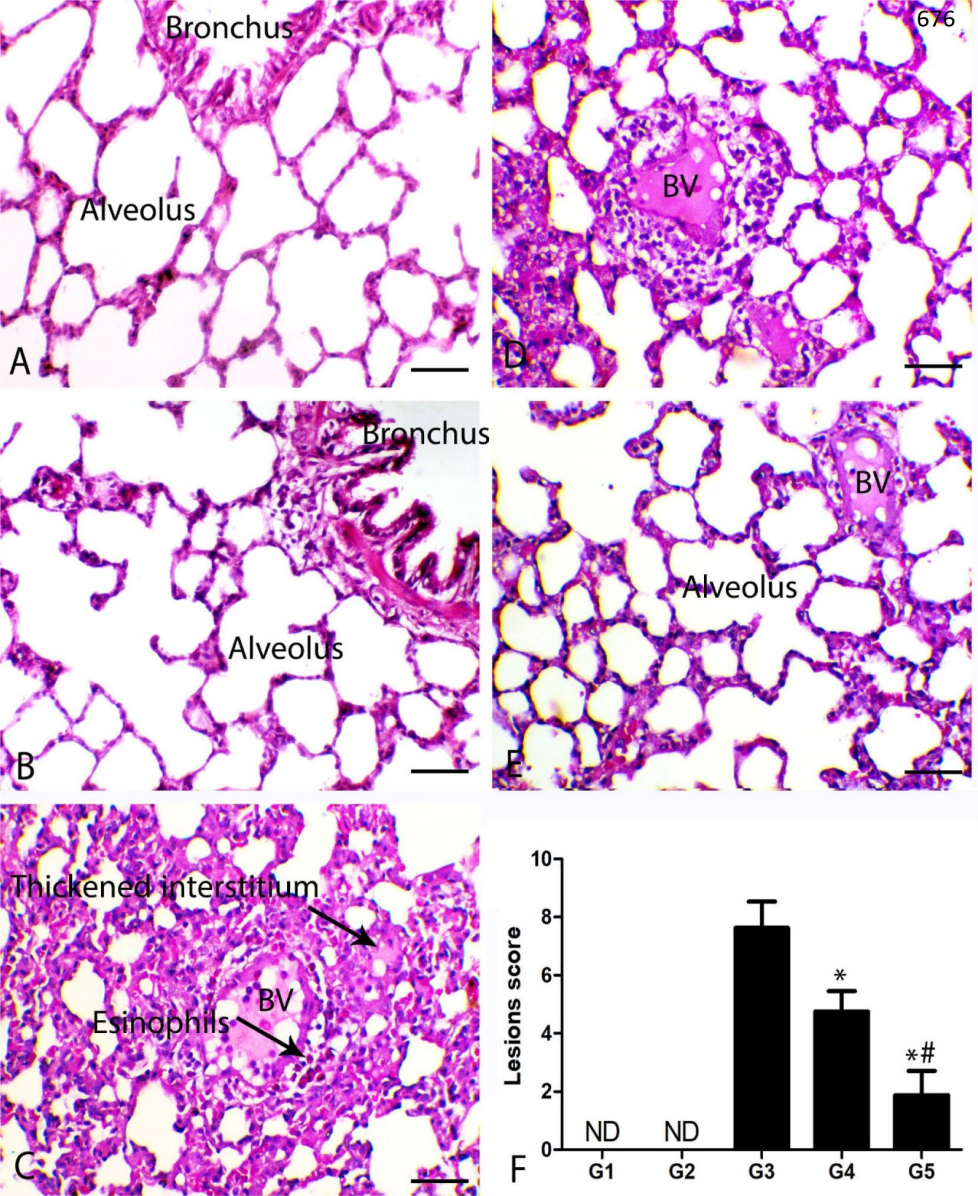
Photomicrograph of the pulmonary sections of the different groups. **(A)** The lung of G1 and **(B)** lung of G2 showing normal alveoli and bronchi. **(C)** Lung of G3 showing interstitial pneumonia associated with perivascular inflammatory cells infiltration mostly eosinophils, BV indicates blood vessels revealing damage of its wall. **(D)** Lung of G4 showing decrease both pneumonia and perivascular inflammatory cells infiltration. **(E)** Lung of G5 showing thin alveolar septa and with marked increase the alveolar spaces. H&E stain bar = 50 µm. **(F)** Quantitative lesion score showing marked decrease the pulmonary lesions, * indicates significe in comparing with G3 (P < 0.005) and # indicates significance in comparing with G4 (P < 0.005).

The lungs of control animals showed normal alveoli and bronchi. Moreover, the lungs of normal animals treated with thymoquinone revealed normal pulmonary tissues. The malathion inhalation revealed severe pulmonary tissue injury. The main pathological feature was interstitial pneumonia. The inflammatory lesions were mostly extended from perivascular to interalveolar interstitial tissues. The blood vessels showed a marked decrease in the tunica media and endothelial cell hypertrophy associated with perivascular oedema and hemorrhage. The perivascular areas showed marked inflammatory cell infiltration, mostly eosinophils and mononuclear cells as lymphocytes and macrophages. The alveolar spaces were markedly decreased associated with interstitial fibroplasia and hypertrophy, and hyperplasia of the pneumocyte type 2 cells. The bronchial tree showed the feature of catarrhal inflammation accompanied by severe desquamation of the bronchial lining cells, regenerative hyperplasia of the lining epithelium, and peribronchial infiltration of lymphocytes, macrophages, and with plenty of eosinophils and basophils. The diseased animals treated with thymoquinone showed a marked decrease in the pneumonic and allergic features in a dose-dependent manner. Malathion and a low dose of thymoquinone showed limited perivascular infiltration of inflammatory cells mostly lymphocytes and macrophages. The interstitial reaction was also decreased with a marked increase in the alveolar spaces. The high dose of the thymoquinone noted a marked decrease of perivascular and interstitial reaction, and most of the alveoli and bronchi were patent without any noticeable inflammation. Quantitative scoring of the histopathological lesions revealed a marked decrease in the lesion score in both thymoquinone-treated groups (p ≤ 0.005) (Fig. 2).

**Figure 3:**
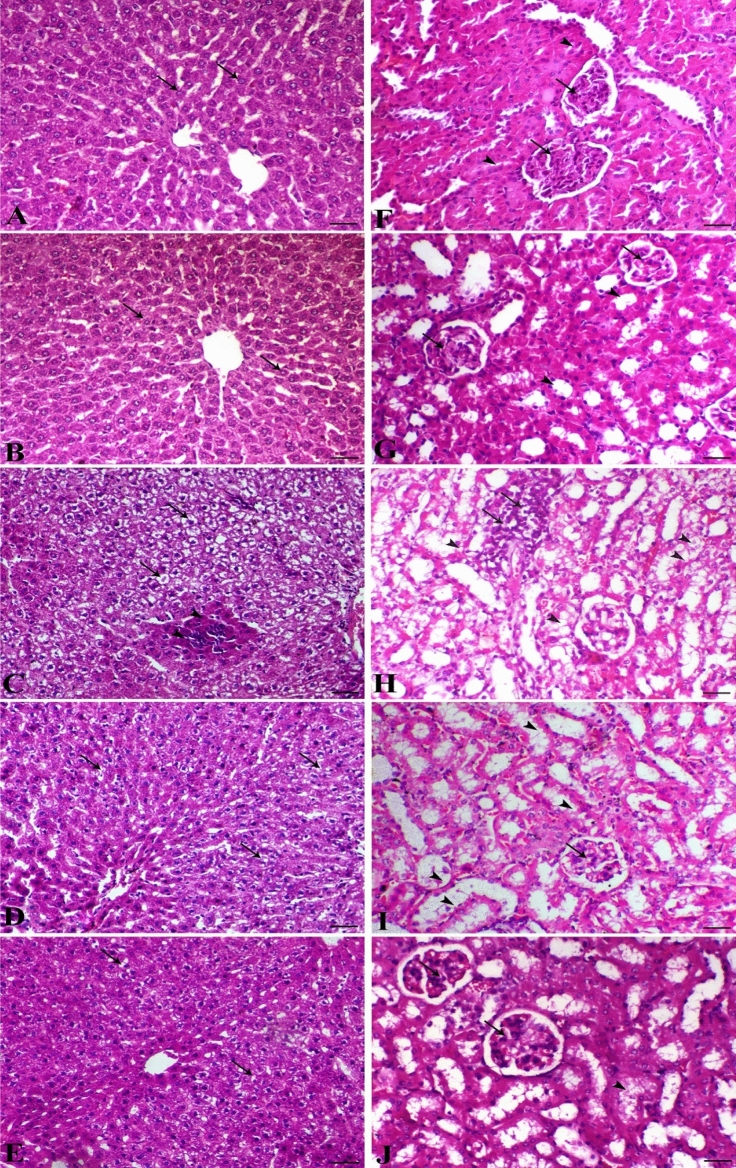
Hepatic **(A–E)** and renal sections **(F–J) **of different treated groups. **(A)** Liver of control group (arrows indicate normal hepatocytes); **(B)** liver of thymoquinone group (arrows indicate normal hepatocytes); **(C)** liver of malathion group (arrows indicate severe hepatic vacuolation and arrowheads indicate focal area of coagulative necrosis); **(D)** liver of malathion + thymoquinone (25 mg/kg) (arrows indicate marked decrease of hepatic vacuolation); **(E)** liver of malathion + thymoquinone (50 mg/kg) (arrows indicate marked decrease of hepatic vacuolation), H&E, bar = 50 µm. **(F)** Kidney of control group (arrows indicate normal glomeruli and arrowheads show normal renal tubules); **(G)** kidney of thymoquinone group (arrows indicate normal glomeruli and arrowheads show normal renal tubules); **(H)** kidney of malathion group (arrows indicate focal interstitial lymphocytic infiltration and arrowheads indicate marked renal tubular epithelium vacuolation); **(I)** kidney of malathion + thymoquinone 25 mg/kg (arrows indicate marked decrease of renal degeneration); **(J)** kidney of malathion + thymoquinone (50 mg/kg) (arrowheads indicate marked decrease of renal tubular epithelium vacuolation and arrow shows normal glomerulus), H&E, bar = 50 µm.

The systemic pathological lesions induced by malathion inhalation upon the hepatic and renal tissues were also evident as illustrated in Fig. 3. The liver of control and sham animals showed normal hepatocytes in a cord-like manner around the central vein. The liver of the control group revealed a severe degree of diffuse hepatic hydropic vacuolation accompanied by a focal area of coagulative necrosis. The malathion-inhaled rats revealed a marked decrease of degenerative and necrotic changes in a dose-dependent manner. The kidney of control and thymoquinone groups showed normal renal glomerular and tubular structures. Malathion group revealed marked tubular degeneration associated with interstitial nephritis features with remarkable mononuclear inflammatory cells infiltration mostly lymphocytes and macrophages. The diseased animals treated with thymoquinone revealed a marked decrease in the tubulo-interstitial degenerative and inflammatory lesions in a dose-dependent manner.

### Mast cell immunostaining

**Figure 4:**
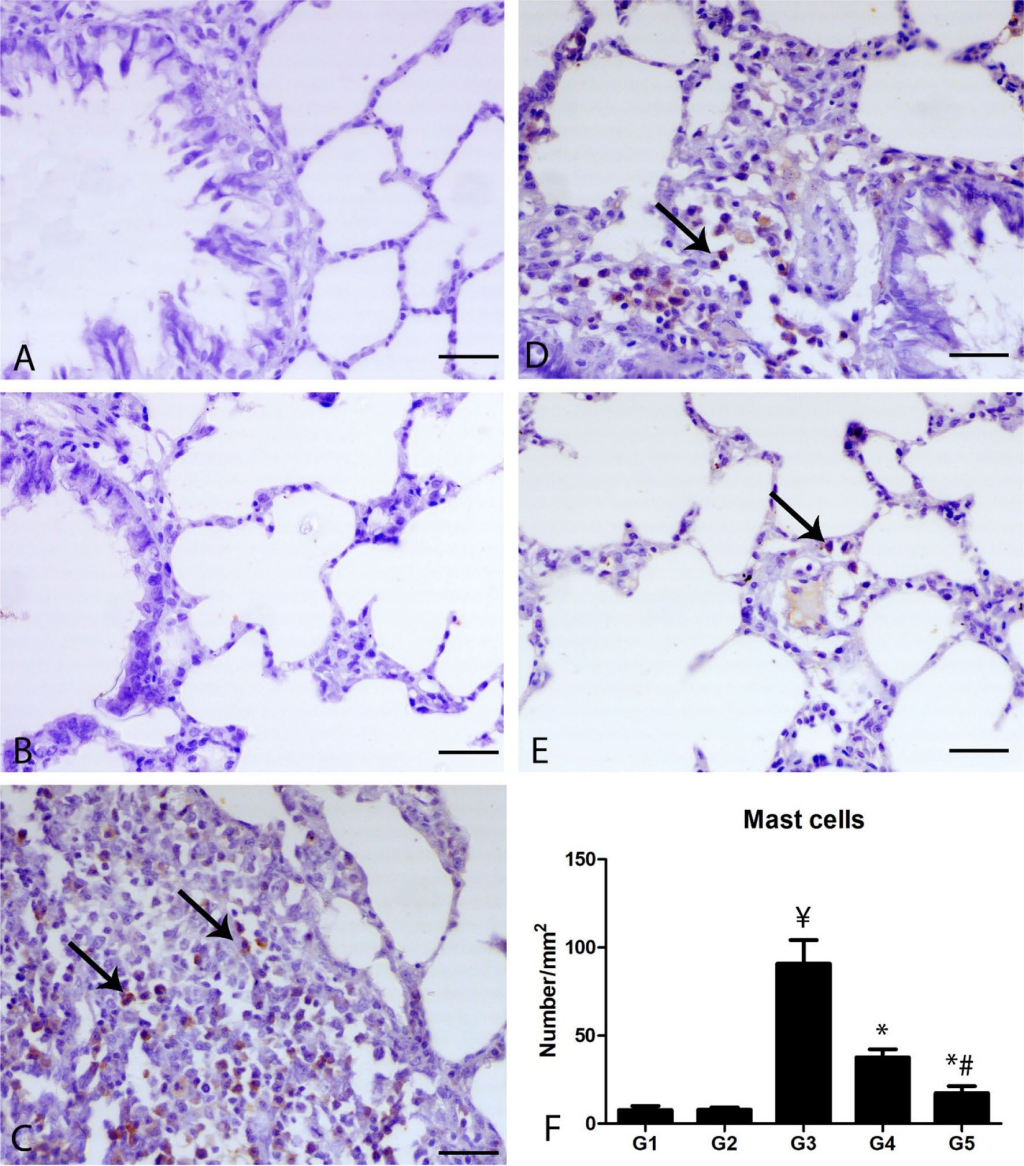
Photomicrograph of the pulmonary sections of the different groups immunostained with C-KIT antibody. **(A)** The lung of G1 and **(B)** lung of G2 showing scarcely expression of C-KIT antibody within the pulmonary tissues. **(C)** Lung of G3 showing numerous C-KIT positive mast cells (arrows). **(D)** Lung of G4 showing decrease of C-KIT positive mast cells. **(E)** Lung of G5 showing marked decrease C-KIT positive mast cells, bar = 50 µm. **(F)** Quantitative score of the number of C-KIT positive mast cells. ¥ indicates significance in comparing with G1 (P < 0.001), * indicates significance in comparison with G3 (P < 0.001) and # indicates significance in comparison with G4 (P < 0.05).

C-KIT (CD117) antibody was used for detection of mast cells within the pulmonary tissues (Fig. 4). The control groups revealed a scanty number of mast cells. Group 3 showed a marked presence of C-KIT positive mast cells, mostly peribronchial and perivascular in comparison with the control group (P ≤ 0.001). Groups 4 and 5 demonstrated a marked decrease in the number of positive mast cells around the airways and the blood vessels in comparison with group 3 (P ≤ 0.001).

### Survivin immunohistochemistry

**Figure 5:**
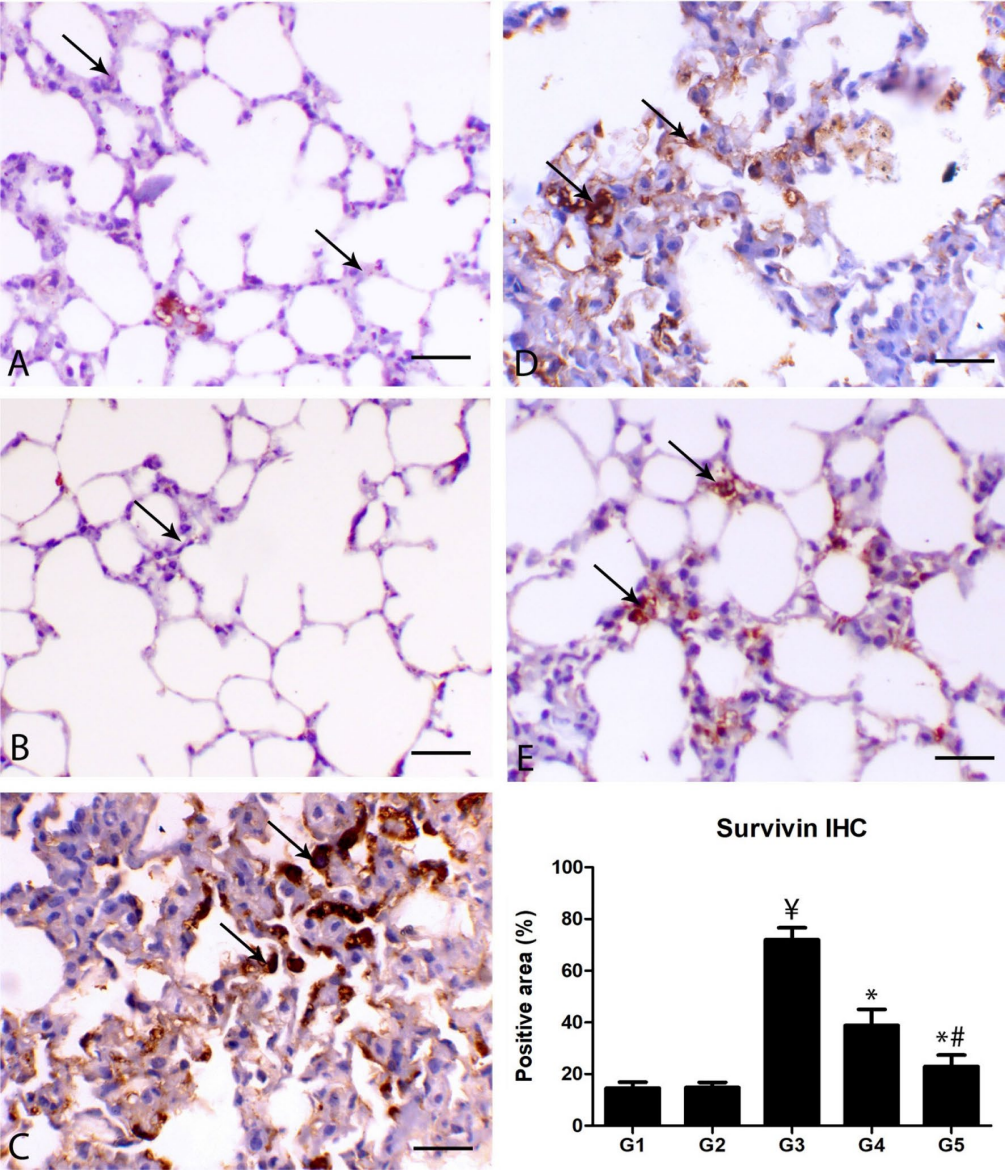
Photomicrograph of the pulmonary sections of the different groups immunostained with surviving antibody. **(A)** The lung of G1 and **(B)** lung of G2 showing mild expression of surviving within the alveolar cells. Normal alveoli and bronchi. **(C)** Lung of G3 showing marked expression within the proliferated alveolar cells mostly alveolar type II. **(D)** Lung of G4 showing decrease of survivin expression within the alveolar cells. **(E)** Lung of G5 showing marked decrease of survivin expression within the alveolar cells, bar = 50 µm. **(F)** Quantitative score of the percent of positive areas showing survivin expression. ¥ indicates significance in comparing with G1 (P < 0.005), * indicates significance in comparison with G3 (P < 0.005) and # indicates significance in comparison with G4 (P < 0.005).

The survivin immunostaining of the lungs of the control animal showed slight within the alveolar cells and moderate expression with the bronchial lining epithelium. A similar reaction was noticed with the same group. In the lungs of malathion-inhaled animals, marked expression of survivin within the alveolar and bronchial lining epithelial cells consistent with the hypertrophied pneumocyte type 2 alveolar cells and the regenerative bronchial lining cells. Diseased animals treated with thymoquinone showed a marked decrease in the percent of survivin immunostaining within the pulmonary tissues in a dose-dependent manner in comparison with the malathion treated group (P ≤ 0.001) (Fig. 5).

### Pulmonary mRNA SP-D expression

**Figure 6:**
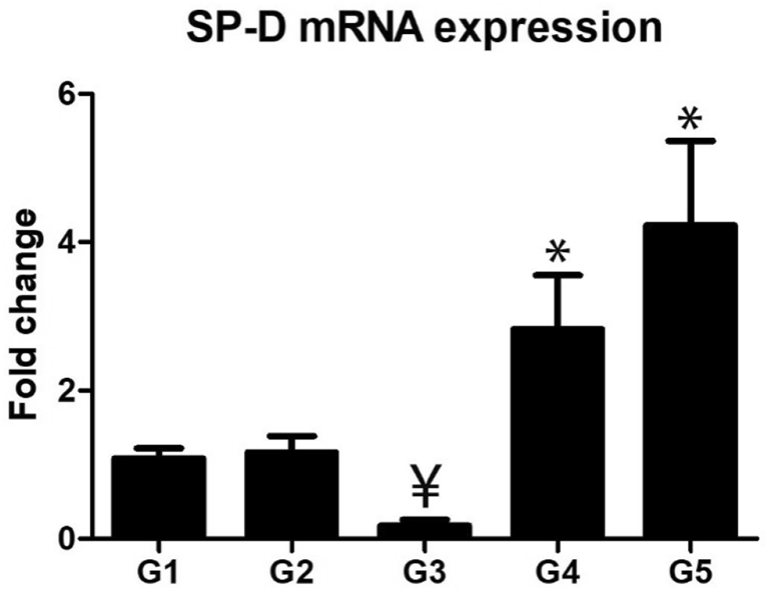
Relative expression of SP-D within the lung tissues which decreased in G3 and markedly increased in G4 and G5. Sign ¥ indicates significance in comparing with G1 (P < 0.005) and sign * indicates significance in comparison with G3 (P < 0.005).

As shown in Fig. 6, SP-D expression was markedly decreased in malathion inhaled animals in comparison with the control group (p ≤ 0.05). In comparison with malathion groups, the diseased rats treated with thymoquinone showed a marked increase of SP-D gene expression within the lung tissues, and marked elevation was noted in animals treated with a high dose of thymoquinone (p ≤ 0.005).

## Discussion

It is noteworthy that respiratory failure is the prime mover of deaths caused by acute OPs toxicity^25^. Haematological blood profiles may provide valuable information on the organism's internal environment^26^. In the present study, malathion inhalation badly affected the blood hematology of the rats. These results are compatible with Kanu et al.^27^ and Ghazy et al.^28^ who reported that other OPs are associated with a significant decrease in the hematological parameters, especially RBCs, hemoglobin, and platelet counts, and with a significant increase in the WBCs count. Moreover, Ghazy et al.^28^ reported decreases in erythrocytes could be as a result of either direct erythrocytes destruction by the pesticide or indirect through its bad effects on the bone marrow. Coles^29^ stated that low packed cell volume promotes reticulocyte released from bone marrow, which increases the MCV. The increment in the MCV and reduction in the MCHC post malathion exposure suggested either haemolytic or hemorrhagic anemia. The obtained results suggested the incidence of macrocytic hypochromic anemia, Where insecticide can initiated erythrocytes destruction as it enhanced the production of reactive oxygen species (ROS), which had an unfavorable effect on erythrocytes membrane. Kalender et al.^30^ attributed the occurrence of macrocytic hypochromic anemia to either interference of the malathion with hemoglobin synthesis or reduction in the RBC life span. The platelet count reduction may be caused by the bone marrow depression by free radicals, which decrease PLT production or depress thrombopoiesis^31^. These adverse effects on the hematological parameters were greatly ameliorated by TQ supplementation. These results are consistent with Ghazy et al.^28^, the alleviation of anemia induced by diazinon toxicity by TQ administration. The improved hematological parameters may be attributed to the ameliorative effect of TQ against free radicals caused by malathion inhalation^32,33^. Malathion was also associated with the inflammation either directly through pro-inflammatory cytokines or subsequently due to the production of the free radicals that leads to the formation of MDA^34^. Inflammation was manifested by a significant increase of leukogram in the malathion inhalation group. These results were correlated well with the histopathology results that appeared by marked perivascular and peribronchial inflammatory cell infiltration. This inflammatory process improved by TQ, which has anti-inflammatory properties^35^.

Liver damage as a result of toxicity or alterations in the membrane architecture of the liver cells can be diagnosed by measuring ALT and AST activities^36,37^. On the other hand, there are several parameters related to renal diseases, most of them deliver information only when renal damage is well established, as is the case for serum creatinine^38^. The results of serum biomarkers of the liver and kidney tissue damaged were statistically affected by malathion inhalation. These results may be attributed to the oxidative damage caused by malathion inhalation on the liver and kidney tissues. These results are in agreement with Al-Attar^39^ and Jabbar et al.^40^ who reported a significant increase in the serum activities of AST, ALT, and ALP and concentration of the urea and creatinine. Moreover, Kanu et al.^27^ recorded the toxic effects of dichlorvos on the liver and kidney of the rats. Abouelghar et al.^41^ reported hematological and biochemical alterations induced by sub-acute exposure to the fipronil in albino mice. These results were confirmed by the results of the liver and kidney histopathology. The histopathological results of the hepatic and renal tissues as a result of malathion inhalation are constant with the result of Mamun et al.^42^ in the mice. Moreover, Selmi et al.^43^ reported severe damages in hepatic and renal tissue in prepubertal male mice treated with malathion. Thus, malathion inhalation induced deterioration in the liver and kidney function, which improved by TQ supplementation. This improvement may be a result of the antioxidant effect of TQ^32^. These results are in harmony with El-Sheikh et al.^44^ who reported that TQ has hepatorenal protection against methotrexate-induced toxicity in the rats through antioxidant, anti-inflammatory, and anti-apoptotic mechanisms. Moreover, Ghazy et al.^28^ reported the protective effect of TQ against the hepatotoxic effect of diazinon in male rats. In addition, Aboubakr et al.^45^ and Farid et al.^46^ recorded the antioxidant protective effect of the natural substances against tilmicosin and Carbon tetrachloride toxicity in the rats, respectively.

All the hematological results in malathion and/or TQ groups were co-related with the results of the histopathology, immunohistochemistry, and gene expression in the lung tissues. The lung of the malathion group showed bronchial and alveolar obliterative lesions associated with marked inflammatory cell infiltration either peribronchial or interstitial. The interesting finding was the allergic features associated with inflammatory cell infiltration as eosinophils and mast cells, macrophages, and lymphocytes. There was a great relationship between the development of asthma and eosinophils infiltration, which may be associated with the production of cytokines responsible for the development of type II sensitivity^47^. Interestingly, malathion is increased basophils, activated mast cell degranulation process, and potentiated macrophages^48^. Moreover, it was noticed that most of the pulmonary blood vessels showed atrophy and loss of the media and endothelial cells, respectively. This might clarify the thrombotic activity of the organophosphorus compounds^49^. The adverse effect of the malathion inhalation was mitigated by TQ supplementation in a dose-dependent manner. The present results are going with Al-Gabri et al.^50^ that noticed that TQ alleviates the lipopolysaccharides-induced lung injury through the reduction of inflammatory edema, thickening of interalveolar septa, hypertrophy of the smooth muscles around the blood vessels and airways, and hyperplasia of the bronchial associated lymphoid tissue. Moreover, TQ has potent anti-inflammatory, spasmolytic, and bronchodilator effects^51^. These explained to increase the pulmonary alveolar spaces in diseased rats treated with TQ. Malathion was associated with marked regenerative hyperplastic changes of type 2-alveolar epithelial cells. Most of the pulmonary toxicants are accompanied by hypertrophy and hyperplasia of type 2 epithelial cells^52,53^. Where the survivin expression increased in the malathion inhalation group. Survivin is a member of the inhibitor of apoptosis protein (IAP) family, regulates mitosis, and chromosome segregation^54,55^. Survivin is mostly undetectable in the normal adult differentiated tissues^56^ and highly expressed in the undifferentiated cells as a notice in many human tumors^57,58^. Although, increase tissue damage may be associated with suppression of antiapoptotic genes including survivin^59,60^. However, it was reported an interesting dignity of the survivin expression associated with a chronic pulmonary injury, which mostly associated with regenerated alveolar cells and infiltrated inflammatory cells. In addition, the results support the cytoprotection role of the survivin against pulmonary^54–61^.

Alveolar type II epithelial cells were the main source of different pulmonary surfactant SP-A, SP-B, SP-C, and SP-D. There was a marked association of increased serum SP-D and progression of chronic obstructive pulmonary disease (COPD), therefore, it has been committed as a biological indicator of COPD status^62^. Malathion group revealed a significant decrease of SP-D mRNA. While treatment of the diseased rats with TQ markedly elevated the SP-D mRNA. It is noteworthy that SP-D acts allergy antagonist through decreased IgE secretion, controlled cytokines produced by Th2, and decreased eosinophilia that mostly abrogates pulmonary inflammation^63,64^. From the aforementioned results, it had been concluded that chronic exposure of malathion inhalation resulting in pulmonary lesions mimicked allergic pneumonia that associated with high serum IgE level, eosinophils, and mast cell tissue infiltration, and low pulmonary surfactants. The administration of TQ markedly ameliorated the malathion-induced pulmonary allergy and obstructive lesions. Therefore, thymoquinone administration could be beneficial for insecticides applicators in crop fields to season.

The current study was approved by the institutional animal care and use committee Kafrelsheikh University, Egypt, 33516.

## Data Availability

All data available on the behave of the corresponding author.

## References

[1] El-Kott AF, Bin-Meferij MM (2008). Influence of green tea on haematological and lung histological disorders induced by malathion in rats. Res. J. Environ. Toxicol..

[2] Binukumar, B. K., & Gill, K. D. Chronic exposure to pesticides-neurological, neurobehavioral and molecular targets of neurotoxicity. in *Pesticides in the Modern World-Effects of Pesticides Exposure*, 3–20 (2011).

[3] Galloway T, Handy R (2003). Immunotoxicity of organophosphorous pesticides. Ecotoxicology.

[4] Noaishi MA, Afify MM, Allah AA (2013). Study the inhalation exposure effect of pesticides mixture in the white rat. Nat. Sci..

[5] EXTOXNET. *Extension Toxicology Network, Pesticide Information Profile, Chlorpyrifos*. http://pmep.cce.cornell.edu/profiles/extoxnet/carbaryl-dicrotophos/chlorpyrifos-ext.html#39. Accessed 8 Apr 2013 (1993).

[6] Ahmed, R. S., Seth, V., & Banerjee, B. D. Influence of dietary ginger (*Zingiber officinales* Rosc) on antioxidant defense system in rat: Comparison with ascorbic acid. (2000). 11116533

[7] Ruckmani A, Nayar PG, Konda VGR, Madhusudhanan N, Madhavi E, Chokkalingam M, Sundaravalli S (2011). Effects of inhalational exposure of malathion on blood glucose and antioxidants level in Wistar albino rats. Res. J. Environ. Toxicol..

[8] Hectors TLM, Vanparys C, Van Der Ven K, Martens GA, Jorens PG, Van Gaal LF, Covaci A, De Coen W, Blust R (2011). Environmental pollutants and type 2 diabetes: A review of mechanisms that can disrupt beta cell function. Diabetologia.

[9] Rezg R, Mornagui B, Benahmed M, Chouchane SG, Belhajhmida N, Abdeladhim M, Kamoun A, El-fazaa S, Gharbi N (2010). Malathion exposure modulates hypothalamic gene expression and induces dyslipedemia in Wistar rats. Food Chem. Toxicol..

[10] Beard J, Sladden T, Morgan G, Berry G, Brooks L, McMichael A (2003). Health impacts of pesticide exposure in a cohort of outdoor workers. Environ. Health Perspect..

[11] Khalifa FK, Alkhalaf MI (2020). Effects of black seed and thyme leaves dietary supplements against malathion insecticide-induced toxicity in experimental rat model. J. King Saud Univ.-Sci..

[12] Shao YY, Li B, Huang YM, Luo Q, Xie YM, Chen YH (2017). Thymoquinone attenuates brain injury via an antioxidative pathway in a status epilepticus rat model. Transl. Neurosci..

[13] Darakhshan S, Pour AB, Colagar AH, Sisakhtnezhad S (2015). Thymoquinone and its therapeutic potentials. Pharmacol. Res..

[14] Mahmoud ES, Al-Shahed FAZN, Ouda EA, Al Anany MG (2019). Effect of thymoquinone on the structure of the cerebral cortex of adult male albino rats treated with tramadol. Sci. J. Al-Azhar Med. Fac. Girls.

[15] Banerjee S, Padhye S, Azmi A, Wang Z, Philip PA, Kucuk O, Sarkar FH, Mohammad RM (2010). Review on molecular and therapeutic potential of thymoquinone in cancer. Nutr. Cancer.

[16] Woo, C. C., Hsu, A., Kumar, A. P., Sethi, G., & Tan, K. H. B. Thymoquinone inhibits tumor growth and induces apoptosis in a breast cancer xenograft mouse model: The role of p38 MAPK and ROS. *PloS One* **8**(10) (2013). 10.1371/journal.pone.0075356 PMC3788809 24098377

[17] Abdel-Daim, M. M., Abushouk, A. I., Bungău, S. G., Bin-Jumah, M., El-kott, A. F., Shati, A. A., Aleya, L., & Alkahtani, S. Protective effects of thymoquinone and diallyl sulphide against malathion-induced toxicity in rats. *Environ. Sci. Pollut. Res.* 1–8 (2020). 10.1007/s11356-019-07580-y 31933077

[18] Hamdan AM, Al-Gayyar MM, Shams MEE (2019). Thymoquinone therapy remediates elevated brain tissue inflammatory mediators induced by chronic administration of food preservatives. Sci Rep.

[19] Kilkenny, C., Browne, W. J., Cuthill, I. C., Emerson, M., & Altman, D. G. Improving bioscience research reporting: the ARRIVE guidelines for reporting animal research. *PLoS Biol.* **8**(6) (2010). 10.1371/journal.pbio.1000412 PMC2893951 20613859

[20] Bancroft J, Layton C (2013). The Hematoxylins and Eosin. Bancroft’s Theory and Practice of Histological Techniques.

[21] Yamanel L, Kaldirim U, Oztas Y, Coskun O, Poyrazoglu Y, Durusu M, Cayci T, Ozturk A, Demirbas S, Yasar M, Cinar O, Tuncer SK, Eyi YE, Uysal B, Topal T, Oter S, Korkmaz A (2011). Ozone therapy and hyperbaric oxygen treatment in lung injury in septic rats. Int. J. Med. Sci..

[22] Khalil RM, Abdo WS, Saad A, Khedr EG (2018). Muscle proteolytic system modulation through the effect of taurine on mice bearing muscular atrophy. Mol. Cell. Biochem..

[23] Tian, Y., Li, J., Li, Y., Dong, Y., Yao, F., Mao, J., & Wang, M. (2016). Effects of Bufei Yishen granules combined with acupoint sticking therapy on pulmonary surfactant proteins in chronic obstructive pulmonary disease rats. *BioMed. Res. Int. *(2016). 10.1155/2016/8786235 PMC5028822 27699176

[24] El-Magd MA, Abdo WS, El-Maddaway M, Nasr NM, Gaber RA, El-Shetry ES, Abdelhady DH (2017). High doses of S-methylcysteine cause hypoxia-induced cardiomyocyte apoptosis accompanied by engulfment of mitochondaria by nucleus. Biomed. Pharmacother..

[25] Ojha A, Srivastava N (2014). In vitro studies on organophosphate pesticides induced oxidative DNA damage in rat lymphocytes. Mutat. Res. Genet. Toxicol. Environ. Mutagen.

[26] Savithri, Y., Sekhar, P. R., & Doss, P. J. Changes in hematological profiles of albino rats under chlorpyrifos toxicity. *Int. J. Pharma Bio Sci.* **1**(3) (2010).

[27] Kanu KC, Ijioma SN, Atiata O (2016). Haematological, biochemical and antioxidant changes in Wistar rats exposed to dichlorvos based insecticide formulation used in Southeast Nigeria. Toxics.

[28] Ghazy, E., Mokh, A., Abdelhady, D., Goda, W., & Hashem, E. (2019). The role of thymoquinone in ameliorating the hepatoxic effect of diazinon in male rats. *Slov. Vet. Res.* **56**(22-Suppl).

[29] Coles EH (1986). Veterinary Clinical Pathology.

[30] Kalender Y, Uzunhisarcikli M, Ogutcu A, Acikgoz F, Kalender S (2006). Effects of diazinon on pseudocholinesterase activity and haematological indices in rats: The protective role of Vitamin E. Environ. Toxicol. Pharmacol..

[31] Elsharkawy EE, Yahia D, El-Nisr NA (2013). Sub-chronic exposure to chlorpyrifos induces hematological, metabolic disorders and oxidative stress in rat: Attenuation by glutathione. Environ. Toxicol. Pharmacol..

[32] Hassanein KM, El-Amir YO (2018). Ameliorative effects of thymoquinone on titanium dioxide nanoparticles induced acute toxicity in rats. Int. J. Vet. Sci. Med..

[33] Jrah Harzallah, H., Grayaa, R., Kharoubi, W., Maaloul, A., Hammami, M., & Mahjoub, T. Thymoquinone, the Nigella sativa bioactive compound, prevents circulatory oxidative stress caused by 1, 2-dimethylhydrazine in erythrocyte during colon postinitiation carcinogenesis. *Oxid. Med. Cell. Longev.* (2012). 10.1155/2012/854065 PMC3337608 22570743

[34] Lasram MM, Dhouib IB, Bouzid K, Lamine AJ, Annabi A, Belhadjhmida N, Gharbi N (2014). Association of inflammatory response and oxidative injury in the pathogenesis of liver steatosis and insulin resistance following subchronic exposure to malathion in rats. Environ. Toxicol. Pharmacol..

[35] Fadl SE, El-Habashi N, Gad DM, Elkassas WM, Elbialy ZI, Abdelhady DH, Hegazi SM (2019). Effect of adding Dunaliella algae to fish diet on lead acetate toxicity and gene expression in the liver of *Nile tilapia*. Toxin Rev..

[36] Mani K, Sundaresan K, Viswanathan K (2000). Effect of dietary aflatoxin B1 on the blood constituents in commercial broilers. Indian Vet. J..

[37] Fuchs TC, Hewitt P (2011). Biomarkers for drug-induced renal damage and nephrotoxicity—an overview for applied toxicology. AAPS J..

[38] Ragheb A, Attia A, Eldin WS, Elbarbry F, Gazarin S, Shoker A (2009). The protective effect of thymoquinone, an anti-oxidant and anti-inflammatory agent, against renal injury: A review. Saudi J. Kidney Dis. Transplant..

[39] Al-Attar, A. M. Physiological and histopathological investigations on the effects of α-lipoic acid in rats exposed to malathion. *BioMed Res. Int.* (2010). 10.1155/2010/203503 PMC2864892 20454535

[40] Jabbar A, Khawaja SA, Iqbal A, Malik SA (1990). Effect of malathion and methyl-parathion on rat liver enzymes. J. Pak. Med. Assoc..

[41] Abouelghar GE, El-Bermawy ZA, Salman HM (2020). Oxidative stress, hematological and biochemical alterations induced by sub-acute exposure to fipronil (COACH) in albino mice and ameliorative effect of selenium plus vitamin E. Environ. Sci. Pollut. Res..

[42] Mamun MAA, Rahman A, Belal SH, Islam MA, Sarker MEH, Arman MSI, Hoque KMF (2015). Histological study of the effect of malathion on liver and kidney tissues of mice model. Int. J. Pharmaceut. Sci. Res..

[43] Selmi S, Rtibi K, Grami D, Sebai H, Marzouki L (2018). Malathion, an organophosphate insecticide, provokes metabolic, histopathologic and molecular disorders in liver and kidney in prepubertal male mice. Toxicol. Rep..

[44] El-Sheikh, A. A., Morsy, M. A., Abdalla, A. M., Hamouda, A. H., & Alhaider, I. A. Mechanisms of thymoquinone hepatorenal protection in methotrexate-induced toxicity in rats. *Mediat. Inflamm.* (2015). 10.1155/2015/859383 PMC4455533 26089605

[45] Aboubakr, M., Elsayd, F., Soliman, A., Fadl, S. E., El-Shafey, A., & Abdelhiee, E. Y. L-Carnitine and vitamin E ameliorate cardiotoxicity induced by tilmicosin in rats. *Environ. Sci. Pollut. Res.* 1–9 (2020). 10.1007/s11356-020-08919-6 32329006

[46] Farid, A. S., El Shemy, M. A., Nafie, E., Hegazy, A. M., & Abdelhiee, E. Y. Anti-inflammatory, anti-oxidant and hepatoprotective effects of lactoferrin in rats. *Drug Chem. Toxicol.* 1–8 (2019). 10.1080/01480545.2019.1585868 30938206

[47] Drake MG, Lebold KM, Roth-Carter QR, Pincus AB, Blum ED, Proskocil BJ, Nie Z (2018). Eosinophil and airway nerve interactions in asthma. J. Leukoc. Biol..

[48] Rodgers K, Xiong S (1997). Effect of acute administration of malathion by oral and dermal routes on serum histamine levels. Int. J. Immunopharmacol..

[49] Pereska Z, Chaparoska D, Bekarovski N, Jurukov I, Simonovska N, Babulovska A (2019). Pulmonary thrombosis in acute organophosphate poisoning—Case report and literature overview of prothrombotic preconditioning in organophosphate toxicity. Toxicol. Rep..

[50] Al-Gabri NA, Ali AM, AL-Attar ES, Hamed M (2017). Pathological study on the role of thymoquinone in experimentally induced acute lung injury in rats. Zagazig Vet. J..

[51] Isik, A. F., Kati, I., Bayram, I., & Ozbek, H. A new agent for treatment of acute respiratory distress syndrome: Thymoquinone. An experimental study in a rat model. *Eur. J. Cardio-thorac. Surg*. **28**(2), 301–305 (2005). 10.1016/j.ejcts.2005.04.012 15949945

[52] Miller BE, Hook GE (1990). Hypertrophy and hyperplasia of alveolar type II cells in response to silica and other pulmonary toxicants. Environ. Health Perspect..

[53] Jansing NL, McClendon J, Henson PM, Tuder RM, Hyde DM, Zemans RL (2017). Unbiased quantitation of alveolar type II to alveolar type I cell transdifferentiation during repair after lung injury in mice. Am. J. Respir. Cell Mol. Biol..

[54] Scheer A, Knauer SK, Verhaegh R (2017). Survivin expression pattern in the intestine of normoxic and ischemic rats. BMC Gastroenterol..

[55] Li G, Zhang H, Zhao L, Zhang Y, Yan D, Liu Y, Fan X (2019). The expression of survivin in irreversible pulmonary arterial hypertension rats and its value in evaluating the reversibility of pulmonary arterial hypertension secondary to congenital heart disease. Pulmonary Circ..

[56] Sah NK, Khan Z, Khan GJ, Bisen PS (2006). Structural, functional and therapeutic biology of survivin. Cancer Lett..

[57] Altieri DC (2008). Survivin, cancer networks and pathway-directed drug discovery. Nat. Rev. Cancer.

[58] Guha M, Altieri D (2009). Survivin as a global target of intrinsic tumor suppression networks. Cell Cycle.

[59] Dohi T, Okada K, Xia F, Wilford CE, Samuel T, Welsh K, Salvesen GS (2004). An IAP-IAP complex inhibits apoptosis. J. Biol. Chem..

[60] Zhang M, Lin L, Lee SJ, Mo L, Cao J, Ifedigbo E, Jin Y (2009). Deletion of caveolin-1 protects hyperoxia-induced apoptosis via survivin-mediated pathways. Am. J. Physiol.-Lung Cell. Mol. Physiol..

[61] Terasaki Y, Terasaki M, Urushiyama H, Nagasaka S, Takahashi M, Kunugi S, Fukuda Y (2013). Role of survivin in acute lung injury: Epithelial cells of mice and humans. Lab. Invest..

[62] Akiki Z, Fakih D, Jounblat R, Chamat S, Waked M, Holmskov U, Sorensen GL, Nadif R, Salameh P (2016). Surfactant protein D, a clinical biomarker for chronic obstructive pulmonary disease with excellent discriminant values. Exp. Therap. Med..

[63] Kishore U, Madan T, Sarma PU, Singh M, Urban BC, Reid KB (2002). Protective roles of pulmonary surfactant proteins, SP-A and SP-D, against lung allergy and infection caused by Aspergillus fumigatus. Immunobiology.

[64] Ikegami M, Scoville EA, Grant S, Korfhagen T, Brondyk W, Scheule RK, Whitsett JA (2007). Surfactant protein-D and surfactant inhibit endotoxin-induced pulmonary inflammation. Chest.

